# Adipose-derived mesenchymal stem cell seeded Atelocollagen scaffolds for cardiac tissue engineering

**DOI:** 10.1007/s10856-020-06425-2

**Published:** 2020-09-23

**Authors:** Qiong Li, Miaomiao Li, Meng Li, Zhengyan Zhang, Han Ma, Liang Zhao, Min Zhang, Guodong Wang

**Affiliations:** 1 Nursing School, Xinxiang Medical University, Xinxiang, 453003 China; 2 Henan Medical Tissue Regeneration Key Laboratory, Xinxiang Medical University, Xinxiang, 453003 China; 3 Third Affiliated Hospital, Xinxiang Medical University, Xinxiang, 453003 China; 4 School of Life Science and Technology, Xinxiang Medical University, Xinxiang, 453003 China; 5 The Affiliated Cancer Hospital of Zhengzhou University, Zhengzhou, 450008 China

## Abstract

ADMSCs were isolated from subcutaneous adipose tissue, characterized and cultured in vitro. GFP-labeled ADMSCs can grow and proliferate well on the Atelocollagen scaffolds, and induced by 5-aza the cells can differentiate into cardio-like cells. 3D cultured ADMSCs on Atelocollagen scaffolds were transplanted into mice ischemia myocardium, and have good biocompatibility with host cardio tissue.

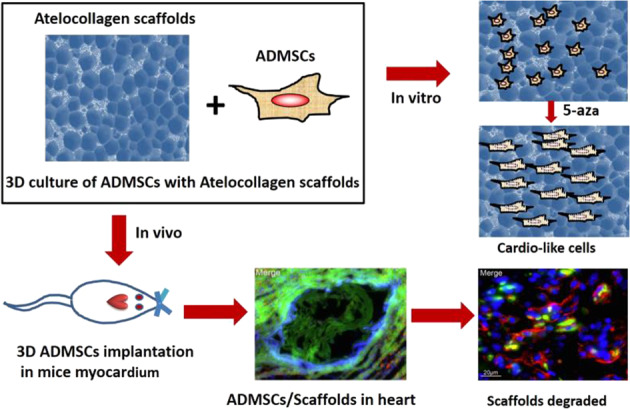

## Introduction

Over the past decades, a variety of stem cells have been used in clinical trials. And again, stem cell transplantation, has been a new method for the treatment of myocardial infarction (MI), a severe clinical condition. In recent years, three types of biological materials have mainly been used for myocardial tissue engineering research: natural materials, synthetic materials, and composite materials. Natural materials mainly include collagen, coral, amino dextran, chondroitin sulfate, gelatin, sodium alginate, fibrin gel, etc. [1–3]. Synthetic materials refer to polyurethane, polyglycolic acid (PGA), polylactic acid (PLA), polyhydroxybutyrate, etc. [4–6]. Composite materials represent chitosan, polylactic acid and multi-walled carbon nanotube fibers, etc. [7]. Ideal myocardial tissue engineering scaffolds materials should be characterized by: non-toxic and good biocompatibility; easy to form 3D structure; high porosity; good surface activity for cell adsorption; biodegradability and plasticity.

Atelocollagen is presently found to be equipped with the abovementioned characteristics and can be used as a tissue filler and for intervertebral disk regeneration culture scaffolds [8]. According to our previous research, Atelocollagen scaffolds could be perfectly combined with neonatal and adult rat cardiomyocytes. In addition, pulsatile 3D myocardial tissue blocks were cultured [9].

Compared to mesenchymal stem cells (MSCs) from other sources, adipose tissue-derived mesenchymal stem cells (ADMSCs) can be isolated easily [10]. ADMSCs have a wide variety of applications, including tissue engineering, regenerative medicine and cell therapy. According to our research, ADMSCs can differentiate into cardiomyocytes, and over-expression of a cocktail of factors can induce ectopic heart formation and program cardiogenesis in ESCs [11]. However, the biocompatibility of ADMSCs and Atelocollagen scaffolds and the differentiation ability of ADMSCs on Atelocollagen scaffolds are not clear.

In the present research, ADMSCs are implanted on Atelocollagen scaffolds for 3D culture, and inducing the cells to differentiate into cardiomyocytes. Biocompatibility of the Atelocollagen scaffolds with ADMSCs and culturing 3D myocardial tissue for myocardial tissue engineering are further explored.

## Materials and methods

### Animals

C57BL/6 mice were provided by Experimental Animal Center of Xinxiang Medical University (Xinxiang, China) and housed at constant temperature and humidity, with a 12-h light and dark cycle, and fed with food and water ad libitum. All protocols were prepared in accordance with provisions of the Committee for Experimental Animals and approved by the Ethics Committee for Animal Research of the Xinxiang Medical University.

### Culture and surface markers test of ADMSCs

The subcutaneous adipose tissue was separated and extracted from the inguinal region. The adipose tissue was washed 3 times and cut into small pieces of 0.5–1 mm^3^. The adipose tissue, added with a double volume of 0.1% collagenase I, was put into the incubator for 10 min. Beat it gently several times in the clean bench and repeat the procedures 3 times. Two volumes of conventional complete medium were used to neutralize the collagenase activity. Filter by 200 strainer mesh, and centrifuge at 1200 r/min for 5 min. The cell suspension was put into a 25-cm^2^ culture flask for 1 h before transference to a new culture flask. The cells were then continuously purified by liquid exchange and passage.

The third-generation ADMSCs were collected at a density of 10^6^/ml and incubated with rabbit anti-mouse CD29-FITC antibody (1:500, BD Company, USA) and CD45-FITC antibody (1:500, BD Company, USA) at 4 °C for 60 min. The surface markers of ADMSCs were detected using flow cytometry.

### Osteogenesis and adipogenesis differentiation ability of ADMSCs

The third-generation ADMSCs were seeded into a six-well plate at 1 × 10^5^/ml. When the cell density reached 70% confluence, the same amount of osteogenic induction reagent was added to each well, and the control group was added with complete culture solution. ADMSCs were induced for 21 days, then washed three times with PBS and fixed with 30% paraformaldehyde for 30–60 min. Distilled water was used to wash three times before 0.1% alizarin red dye solution was added. ADMSCs were then stained with 0.1% alizarin red dye solution in a 37 °C incubator for 30 min and washed three times with PBS before observance under an inverted microscope and taking photographs.

The third generation ADMSCs were inoculated into a six-well plate at 1 × 10^4^/ml when the cells reached 70% confluence. An equal amount of adipogenesis induction reagent was added to each well, and the control group was supplemented with conventional complete medium. At the 14th day, cells were washed with PBS for 3 times, fixed with 4% paraformaldehyde for 5 min, rinsed with 70% isopropanol two times and stained with oil red O before observance under the microscope and taking photographs.

### Labeling ADMSCs with GFP

The third generation ADMSCs were inoculated into a six-well plate at a density of 1 × 10^5^/ml. ADMSCs were infected with AAV-GFP virus at multiplicity of infection (MOI) 5, 10, 25, 50 for 8 h, washed with PBS and cultured in a complete medium containing 10% FBS for 72 h. The green fluorescence and cytotoxicity of the infected cells were observed under a living cell workstation, and the appropriate MOI was selected. Neomycin (G418, 200 μg/ml) was used to purify and screen the GFP transfected cells.

### 3D culture of ADMSCs on Atelocollagen scaffolds

The Atelocollagen is a biological collagen from cattle and has no antigenicity and toxic side effects on organisms (granted by Pro. Yoshinori Kuboki of Hokkaido University) [12]. The Atelocollagen scaffolds were placed at the bottom of the EP tube, the GFP-labeled ADMSCs (1 × 10^6^/ml) were transferred onto the scaffolds and put in the incubator for 4 h. Then the scaffolds were carefully aspirated and placed in a culture dish. The growth and distribution of ADMSCs on the Atelocollagen scaffolds were observed under the living cell workstation at 1 d, 3 d, 7 d.

### Induction of cardiac differentiation of ADMSCs on Atelocollagen scaffolds in vitro

ADMSCs were filled with 70 to 80% of the mesh of the scaffolds, induced with DMEM containing 10-μmol/L 5-azacytidine (5-aza) for 24 h and cultured in complete medium for 2 to 3 weeks. ADMSCs and Atelocollagen scaffolds complex were fixed with 4% paraformaldehyde for 30 min, treated with 0.3% Triton for 10 min and blocked with goat serum for 30 min. Rabbit anti-mouse cardiac troponin T antibody (cTnT, 1:400, Abcam, Shanghai, China) was added and put in a humidified box at 4 °C overnight. PBS was used to rinse 3 times. Cy3 labeled goat anti-mouse immunofluorescent antibody (1:200, Beyotime Biotech, Jiangsu, China) was incubated at room temperature for 1 h. The nucleus was stained with DAPI for 10 min, and observed under a fluorescence microscope. Control groups were incubated with PBS rather than primary antibody.

### 3D culture of ADMSCs with Atelocollagen scaffolds in mice myocardium

GFP-labeled ADMSCs were put on the Atelocollagen scaffolds composition in a dish and placed in the refrigerator at 4 °C. The surgery was performed on the animals anesthetized by 10% chloralose. The heart was slit open at the apex and the cell-collagen scaffolds composition were stuffed into the incision. The position of composites was checked and the wound sutured layer by layer. After intraperitoneal injection of 1.6 million U penicillin, the tracheal intubation was removed and the neck skin was sutured when the mice recovered to spontaneous breathing. Mice of different groups were separately fed in a cage.

Mice were sacrificed by cervical dislocation at 1 d, 3 d, 7 d, 9 d and 14 d after transplantation. The heart was quickly removed and excessive blood was flushed away by ice PBS. After fixation by 4% paraformaldehyde and dehydration in 18% sucrose, it was embedded with OCT. Cryostat sections (8 μm) were mounted on the glass slide, baked at 37 °C for 30 min, and incubated with the primary antibody against cTnT and Cx43 diluted 1:300 in PBS. The sections were then rinsed again in PBS and incubated for 60 min with the goat secondary antibody conjugated to FITC (1:200, Beyotim Biotech Co., Ltd, Jiangsu, China). Control groups were incubated with PBS rather than the primary antibody. The expression of cTnT and Cx43 was observed under a fluorescence microscope.

### Statistical analysis

Data are present as Mean ± SD. Statistical comparisons were made by one-way analysis of variance with the Tukey post test. *P* < 0.05 indicated significant difference.

## Results

### Flow cytometry analyses of cultured ADMSCs surface markers

**Fig. 1:**
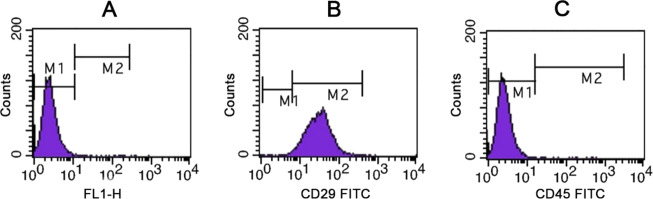
Expression of CD29 and CD45 on the surface of ADMSCs. **a** Control group; **b** CD29-FITC; **c** CD45-FITC

The results of flow cytometry showed that the expression rate of CD29 on the surface of ADMSCs was 94%, but that of CD45 was only 0.19%. It has the internationally-recognized surface molecular characteristics of mesenchymal stem cells, proving that such cells are adipose-derived mesenchymal stem cells (Fig. 1).

### Detection of osteogenic and adipogenic differentiation of ADMSCs

**Fig. 2:**
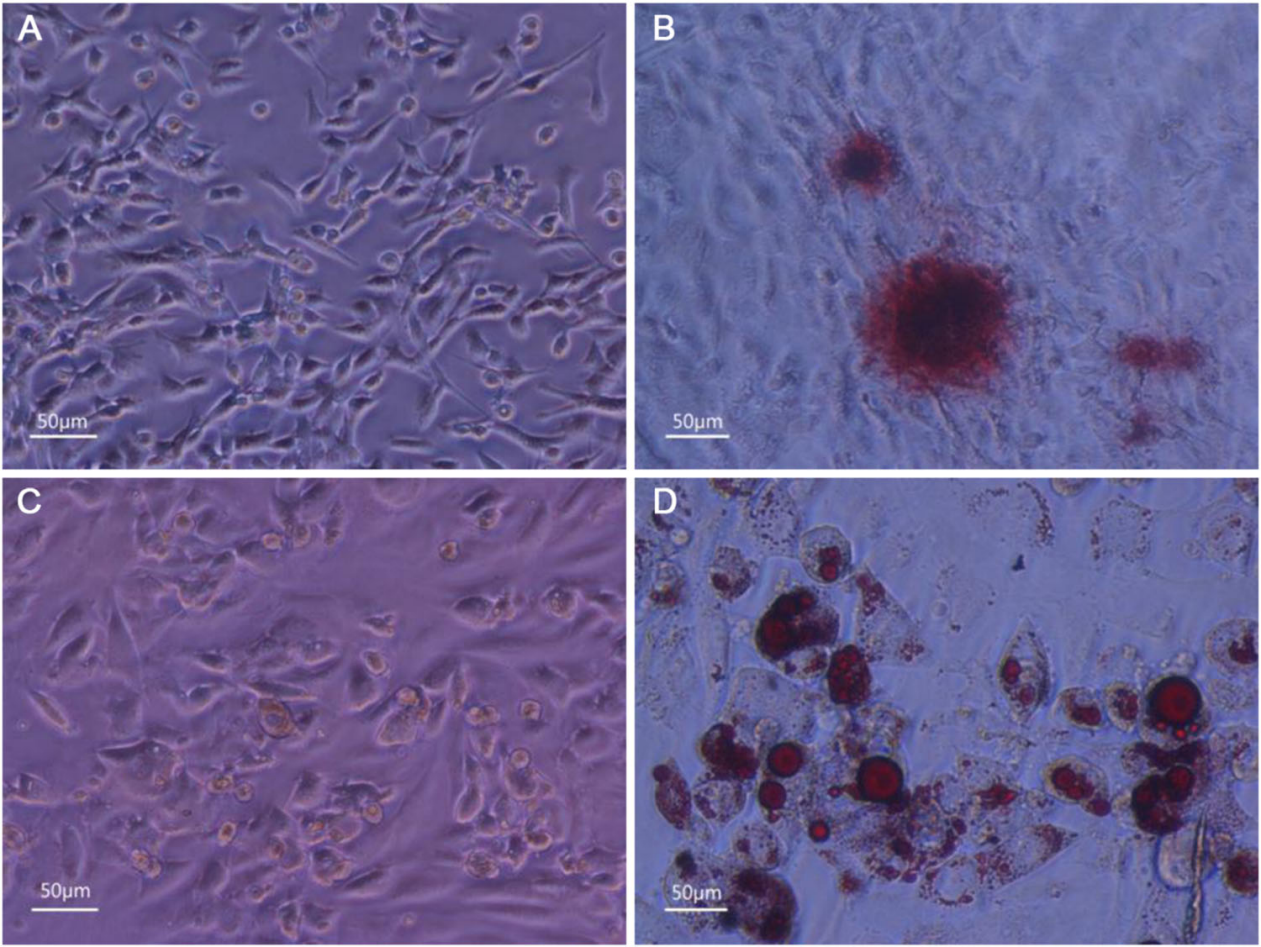
Differentiation and identification of osteogenesis and adipogenicity of ADMSCs. **a** Osteogenesis control group; **b** Alizarn Red staining of the ADMSCs after osteogenically induced for 14 days. **c** Adipogenic induction control group. **d** Oil red O staining after 14 days of adipogenic induction

The size of the third-generation ADMSCs increased gradually in the three days culture under the induction of osteogenic medium. At 14 d, the structure of the small dark brown knots appeared and increased significantly. After 14 days, the red calcified plaque structures were found on the dish when stained by alizarin red. It indicated that ADMSCs had differentiated into bone cells (Fig. 2a, b).

The third-generation ADMSCs produced round lipid vacuoles in the cytoplasm after 14 days induced by adipogenic medium. The morphology was extremely close to that of mature adipocyte, and the oil red O staining was positive, which showed ADMSCs had differentiated into adipocytes (Fig. 2c, d).

### The ADMSCs are compatible with Atelocollagen scaffolds

**Fig. 3:**
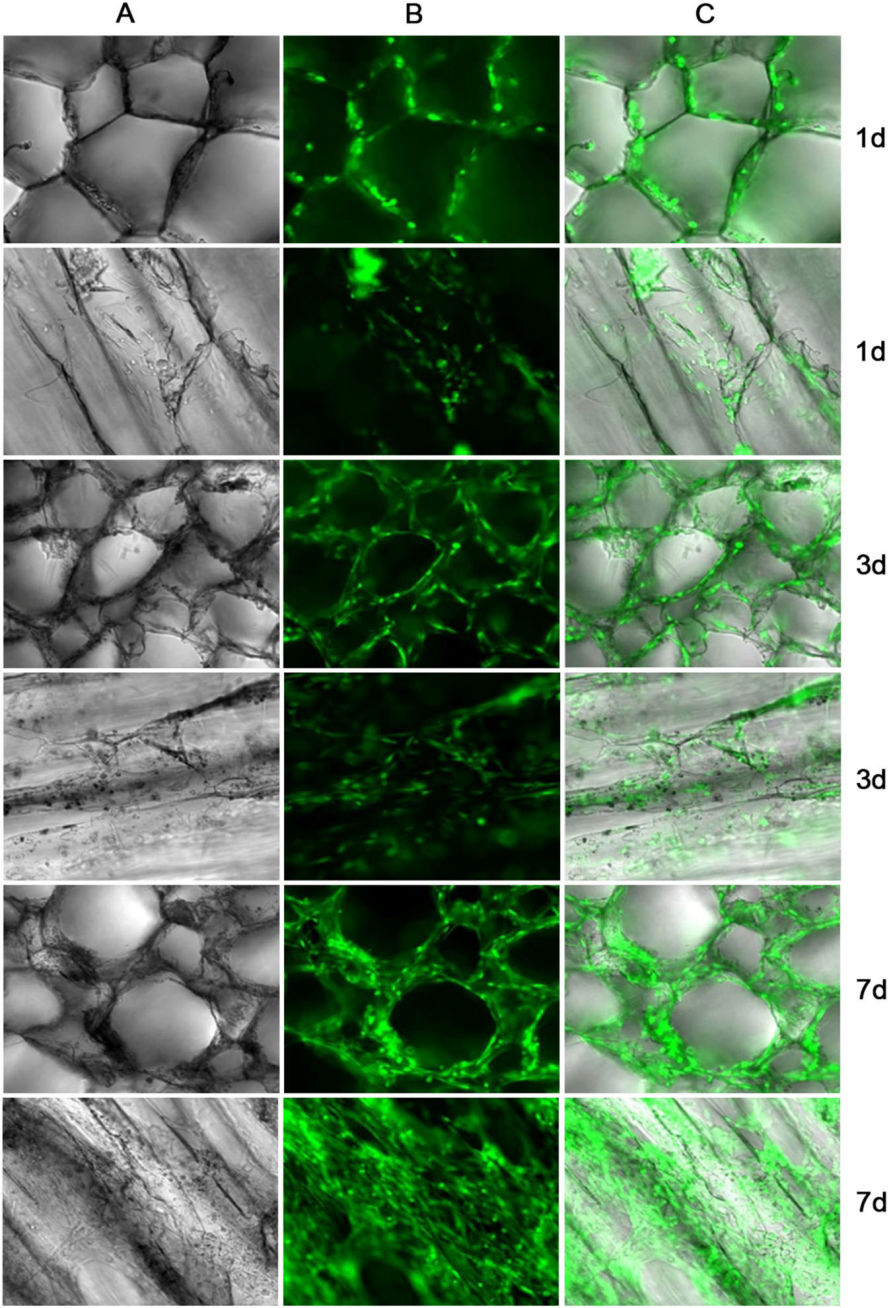
The image of ADMSCs compatible with the Atelocollagen scaffolds. Columns **a**–**c** represents pictures of optical microscope, fluorescence microscope and merged pictures of **a** and **b**. Pictures in Row 1 and 2 are cultured ADMSCs-GFP in the Atelocollagen scaffolds on the 1st day. Pictures in Row 3 and 4 are cultured ADMSCs-GFP in the Atelocollagen scaffolds on the 3rd day. Pictures in Row 5 and 6 are cultured ADMSCs-GFP in the Atelocollagen scaffolds on the 7th day

Proliferation of ADMSCs on the collagen scaffolds of Atelocollagen was evaluated by measuring the fluorescence of GFP labeled ADMSCs. The ADMSCs grew rapidly on the collagen scaffolds of Atelocollagen, and the cells can grow into the mesh of the collagen scaffolds. On the 7th day, about 50% of the mesh of the scaffolds were filled with GFP-labeled ADMSCs (Fig. 3).

### Expression of cTnT on the 5-aza induced 3D cultured ADMSCs

**Fig. 4:**
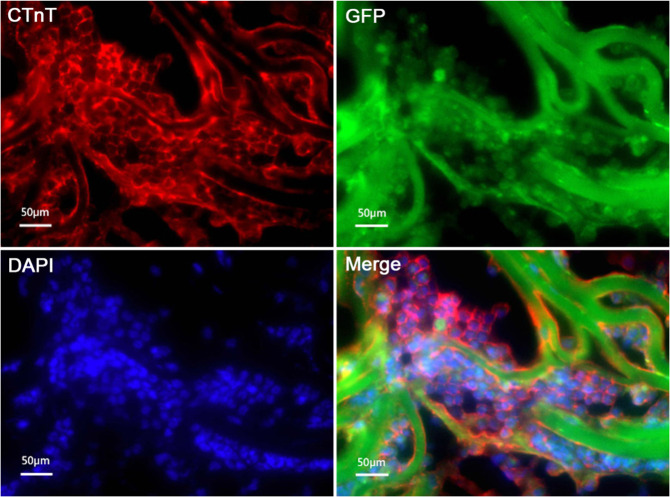
cTnT expression of ADMSCs induced 3 weeks with 5-aza on 3D scaffolds by immunofluorescence staining. **a** Red shows cTnT; **b** green shows GFP; **c** blue shows the nucleus; **d** merge of **a**, **b**, and **c**. Bar = 50 μm (color figure online)

ADMSCs were cultured on 3D Atelocollagen scaffolds. After 3 weeks of 5-aza induction, cTnT immunofluorescence was positive (Fig. 4).

### Effects of ADMSCs implantation on mice myocardium and histological alterations

**Fig. 5:**
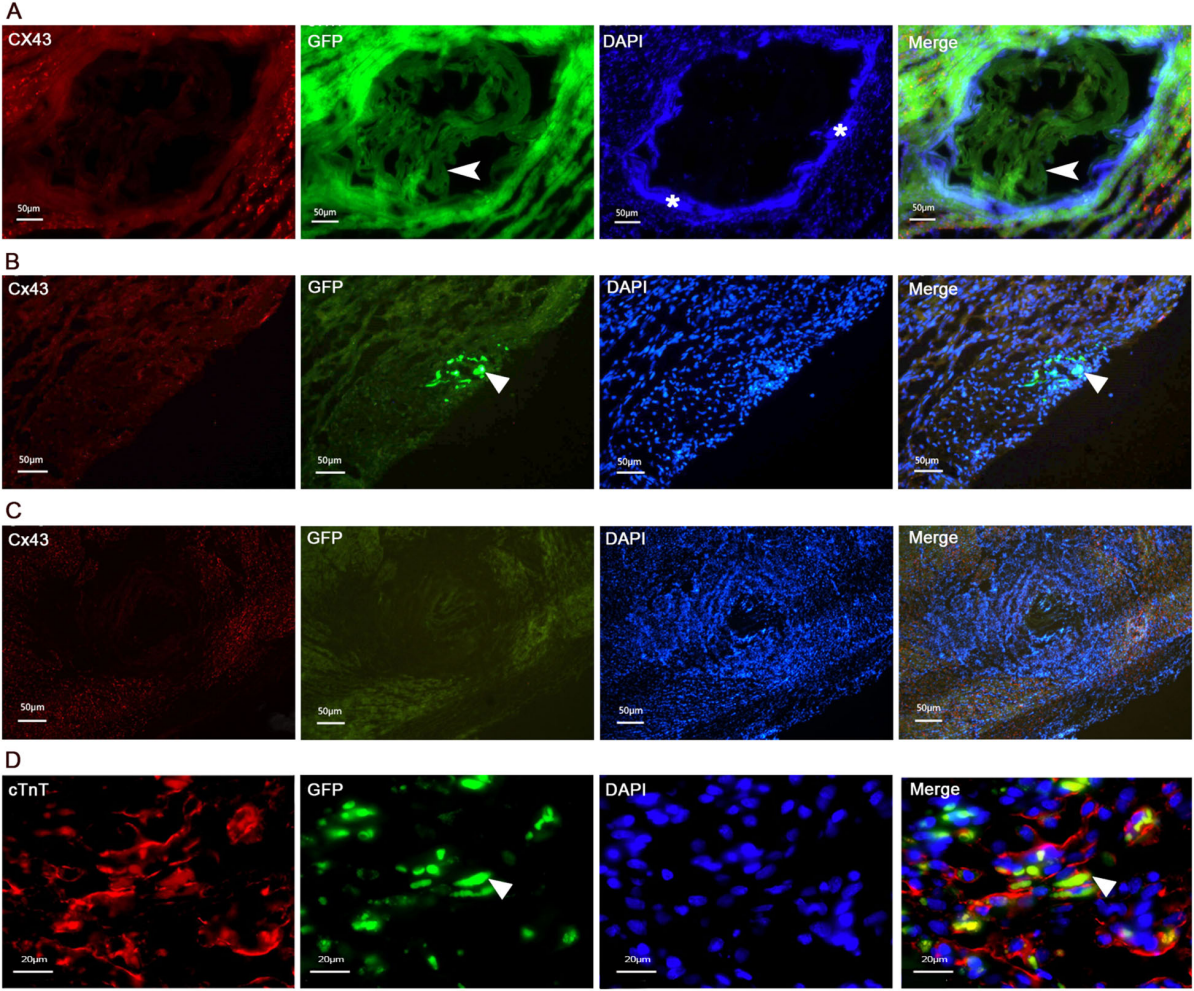
Effects of ADMSCs-GFP transplantation on mice myocardium and histological alterations. **a** Survival cells on scaffolds 3 days after transplantation by immunofluorescence, arrowhead shows the scaffolds, asterisk shows connective tissue; **b** expression of Cx43 in ADMSCs after GFP-ADMSCs based collagen scaffolds transplantation for 7 days, triangle arrow shows the transplanted cells that migrate in the host tissue; **c** Cx43 expression of ADMSCs after seeding on collagen scaffolds for 9 days. **d** cTnT expression of ADMSCs after GFP-ADMSCs based collagen scaffolds transplantation for 9 days. Triangle arrow shows the transplanted cells that differentiates into cardiomyocyte-like cells

Three days after transplantation, the number of nucleus on the scaffolds decreased sharply compared with that before transplantation, which means the ADMSCs grew into the scaffolds. The scaffolds were surrounded by connective tissue, acting like a circle of “isolation zone” (Fig. 5). Most of the transplanted cells were distributed here, some of the transplanted cells passed through the “isolation zone” and migrated to the normal cardiomyocytes of host. On the 7th day and 9th day, we detected the expression of cTnT and Cx43 on transplanted cells by immunofluorescence, and found that the expression of Cx43 on host normal cardiomyocytes was positive. Interestingly, we only found some of the transplanted ADMSCs expressed cTnT, but none of the transplanted ADMSCs expressed Cx43. These results indicated that some of the transplanted ADMSCs had differentiated into cardiomyocyte-like cells. But we did not find any functional or communication relationship between host cardiomyocyte cells and transplanted cells (Fig. 5). On the 9th day, the scaffolds grafted into the myocardium were nearly dissolved, and the original site of the scaffolds were filled with connective tissue. On the 14th day all of the scaffolds disappeared (Fig. 5).

Blue shows cell nucleus; green shows transplanted ADMSCs labeled GFP; red shows Cx43 or cTnT; merge shows a, b, and C simultaneously.

## Discussions

In the latest years, many studies have shown that myocardial tissue engineering may be an ideal method for the treatment of ischemic heart disease such as acute myocardial infarction [13, 14]. It is very important to select the appropriate cells and materials for myocardial tissue engineering. In the present research, Atelocollagen scaffolds were tested as the ADMSCs delivery system and regenerative cardiac therapeutic in a mice model of myocardial infarction. It was found that ADMSCs grew well on Atelocollagen scaffolds and had significantly better survival in the ischemic myocardium.

Previous studies have shown that ADMSCs have plenty of advantages in constructing multicellular lamellae for the treatment of myocardial infarction. On the one hand, ADMSCs have strong proliferation ability and overlapping growth characteristics [15]. On the other hand, ADMSCs can secrete angiogenesis-related factors and promote neovascularization [16–18]. Furthermore, the multi-directional differentiation potential of ADMSCs can accelerate myocardial tissue remodeling and improve cardiac function after myocardial infarction [19]. Some studies have shown that ADMSCs can improve cardiac function after AMI and transplantation [20, 21]. Therefore, ADMSCs play an important role in improving left ventricular remodeling after myocardial infarction. According to this research, ADMSCs have the ability of proliferation and accelerating myocardial tissue remodeling after myocardial infarction. The experimental results were consistent with the identification criteria proposed by Okura [22].

The 3D printing technology has been intensively explored in recent years, which is reconstructed with the imaging data of human anatomy, and other biological and chemical factors are added to the printing material [23]. The biological 3D structure is more accurate, and the regeneration of functional tissue is more effective. The printing technology has been successfully applied in the fields of dentistry, maxillofacial, muscular and skeletal fields. However, its application in cardiac tissue engineering is still under research [24]. Guyette et al. [25] obtained a human acellular heart scaffolds based on decellularization, and filled non-transgenic human induced pluripotent stem cell-derived cardiomyocytes into the heart matrix. As a result, 3D artificial heart tissue is produced, which realizes the construction of human myocardial tissue and lays a solid foundation for the treatment of heart transplantation or cardiovascular disease. Lu et al. [26] encapsulated embryonic stem cells into a thermosensitive chitosan hydrogel and injected them into the myocardium of mice with myocardial infarction for one week. The cardiac function, wall thickness, and neovascular density in the infarct area increased significantly. Nakamuta et al. [27] injected fibrin glue as a carrier for bone marrow cell transplantation into mice infarcted myocardium, and significantly improved cell retention and survival at the injection site compared with the solo cell transplantation group. Holnthoner et al. [28] investigated the ability of Endothelial cells derived from peripheral blood to form vascular structures in co-culture with ADMSCs in a fibrin matrix, adding new insights into co‐culture‐induced vessel formation of outgrowth endothelial cells within a fibrin matrix in an autologous system. Kofidis et al. [29] transplanted mice embryonic stem cells with Matrigel, which could improve left ventricular function better than cells or gel alone group. So the scaffolds used in myocardial tissue engineering should have fine biocompatibility, or else it may cause more damage. In this research, we demonstrated for the first time that ADMSCs can attach, proliferate and differentiate on Atelocollagen scaffolds, suggesting that this biomaterial may be used in 3D printing to print out suitable scaffolds for cardio repair according to infarct size, shape, etc.

To validate the good biocompatibility of the ADMSCs and Atelocollagen scaffolds, we implanted GFP-tagged ADMSCs on Atelocollagen scaffolds, which grew well and proliferated rapidly. The GFP-labeled ADMSCs gradually filled the mesh of the scaffolds, up to ~50% of mesh on the 7th day. The result indicates that the ADMSCs can grow well on Atelocollagen scaffolds, providing possibilities for in vivo experimental studies. The ADMSCs on Atelocollagen scaffolds were consecutively inducted by 5-aza for three weeks, and the expression of cTnT detected by immunofluorescence assay was positive, which indicated that ADMSCs had differentiated into cardiomyocytes and further offered possibilities for in vivo experimental studies.

In addition, because the heart is a highly vascularized organ, the primary tissue construct needs to contract synchronously and spontaneously and the development of its artificial structure is very challenging [30, 31]. In order to establish the ideal myocardial biodegradable scaffolds, a critical balance needs to be reached between the rate of cell tissue formation and the rate of scaffolds degradation. Too early degradation may reduce the integrity of the host tissue or organ and cause further damage to the host. When the scaffolds stay in the host too late, it may prevent cell integration and angiogenesis and lead to scar formation. Many of biodegradable scaffolds have been successfully used in the application of cartilage [32], bladder [33] and blood vessels [34]. In this research, ADMSCs were combined with Atelocollagen scaffolds. On the 9th day, we found the scaffolds grafted into the myocardium nearly dissolved, the formation of connective tissue is a typical reaction to collagen-based biomaterials and can help to resist deformation and rupture of the infarct area. Atelocollagen scaffolds all degraded after 14 days of transplantation, and ADMSCs migrated to normal cardiomyocytes and differentiated into cardiomyocytes. However, we did not find any functional or communication relationship between host cardiomyocyte cells and transplanted cells.

This research paper highlights the ADMSCs seeded on the scaffolds can migrate into host heart tissue and differentiate into cardiomyocytes. Because the heart tissue engineering is complicated, further explorations on the quantity and quality of ADMSCs and supplementary conditions are required.

## Conclusions

We have demonstrated that ADMSCs and Atelocollagen scaffolds have good biocompatibility both in vitro and in vivo. GFP-labeled ADMSCs can differentiate into the myocardial cells and be suitable for the 3D culture of myocardial tissue on the Atelocollagen scaffolds.
